# Responses of Methane Emissions to Different Soil Amendments in Paddy Soil: Soil Properties, Microbial Communities, and Functional Genes

**DOI:** 10.3390/biology15110883

**Published:** 2026-06-03

**Authors:** Qiong Wu, Dalu Deng, Yuwen Zhang, Weiwen Liang, Yifan Li, Yaping Zhang, Yi Wang

**Affiliations:** Guangdong Key Laboratory of Environmental Catalysis and Health Risk Control, School of Environmental Science and Engineering, Institute of Environmental Health and Pollution Control, Guangdong University of Technology, Guangzhou 510006, China

**Keywords:** soil amendment, methane emission, paddy soil, microbial inoculants, anaerobic microcosm

## Abstract

Paddy soils are important contributors to agricultural greenhouse gas emissions, particularly methane, and soil amendments may regulate methane production by altering soil physicochemical properties and microbial methane cycling. However, the effects of different amendment types on methane emissions from anaerobic paddy soils remain uncertain. In this study, an anaerobic microcosm experiment was conducted to evaluate the effect of microbial inoculants, biochar, humic acid, and montmorillonite on CH_4_ and CO_2_ emissions from paddy soil. Changes in acetate concentration, pH, electrical conductivity, microbial community structure, and methane cycling functional genes were further analyzed to explore the underlying mechanisms. The results showed that microbial inoculants had stronger effects on CH_4_ emissions than the other amendments, but their effects were contrasting. The Chabeijian (CB) inoculant significantly increased methane emissions by 100.8%, whereas the Duojun-360 (DJ) inoculant reduced cumulative methane by 57.1%. The stimulation of CH_4_ emissions under Chabeijian was associated with enhanced acetate turnover, enrichment of methanogenic taxa including *Methanosarcina*, *Methanobacterium*, *Methanocella*, and *Methanosaeta*, and a 48.7% increase in *mcrA* abundance. In contrast, Duojun 360 markedly increased soil electrical conductivity, reduced methanogen abundance, decreased *mcrA* abundance by 26.9%, and lowered the *mcrA*/*pmoA* ratio, indicating a shift away from methane production. Although both inoculants increased methanotroph abundance and *pmoA* abundance, methane production remained the dominant factor controlling net CH_4_ emissions. These findings may provide preliminary mechanistic support for the targeted selection of soil amendments to mitigate CH_4_ emissions in rice cultivation by regulating soil properties, methanogenic communities, and the balance between methane production and oxidation.

## Simple Summary

Rice fields release methane, a powerful greenhouse gas that contributes to climate change. Soil amendments are widely used to improve soil quality, but their effects on methane release are not always predictable. This study tested microbial inoculants, biochar, humic acid, and montmorillonite using a soil microcosm experiment that simulated flooded paddy soil. The results showed contrasting effects among amendments. Duojun 360 reduced methane emissions by 57.1%, whereas Chabeijian increased methane emissions by 100.8%. Biochar, humic acid, and montmorillonite had relatively minor effects on methane emissions compared with the two microbial inoculants. The opposite effects of Duojun 360 and Chabeijian were closely related to methane cycling microorganisms and functional genes. Chabeijian enriched methane producing microorganisms, including *Methanosarcina*, *Methanobacterium*, *Methanocella*, and *Methanosaeta*, and increased the methane production gene mcrA. In contrast, Duojun 360 reduced these microorganisms and decreased *mcrA*. Both microbial products increased methane consuming microorganisms, such as *Methylocystis*, and increased the methane oxidation gene *pmoA*, but methane production mainly determined the final methane release. These findings indicate that soil amendments should be carefully evaluated before use in rice fields. This study may help guide the selection of suitable amendments for reducing greenhouse gas emissions from rice cultivation.

## 1. Introduction

Methane (CH_4_) are one of the most significant greenhouse gases (GHGs) contributing to global climate change, with CH_4_ possessing a much higher global warming potential than CO_2_ over a 100-year period [1]. Paddy fields, due to their waterlogged and anaerobic conditions, represent one of the largest anthropogenic sources of CH_4_, accounting for approximately 11% of global CH_4_ emissions [2,3]. Methane emissions from paddy soils are primarily derived from anaerobic microbial processes, including methanogenesis and organic matter decomposition [4,5]. Soil amendments, agricultural management practices, and climate change can regulate these processes by altering microbial communities and soil properties, thus impacting soil methane emissions [6,7,8,9]. Consequently, mitigating methane emissions from rice cultivation while maintaining or improving soil productivity has become a critical challenge for sustainable agricultural development [10].

Soil amendments are widely used to enhance soil fertility, improve crop productivity, and regulate soil physicochemical properties [11,12]. These amendments include a broad range of organic and inorganic materials, such as biochar, compost, silicate fertilizers, clay minerals, microbial inoculants and so on [9,12]. Recent research has primarily focused on their benefits in increasing soil organic carbon (SOC) sequestration, stabilizing organic matter, enhancing nutrient availability, and improving soil structure [13,14]. Moreover, amendments are known to improve soil physical properties such as aeration, water retention, and aggregate stability, which collectively promote plant growth and soil health [15,16]. While the agronomic benefits of these amendments are well-documented, their impact on soil carbon emissions, particularly CH_4_ emissions, have received less attention.

The impact of soil amendments on CH_4_ emissions are largely determined by their interactions with soil microbial communities, carbon cycling and physicochemical properties. For example, microbial inoculants can alter microbial community composition, potentially enhancing methane oxidation by promoting methanotrophs or suppressing methanogens, while also accelerating organic matter decomposition and increasing CO_2_ emissions [17,18]. The organic amendments, including organic fertilizer, biochar and humic substances, can stimulate microbial metabolism and enhance CH_4_ emissions [9,19,20]. However, biochar can also modify soil redox conditions and create surfaces for microbial colonization, which may suppress CH_4_ emissions by favoring the presence of alternative electron acceptors [21]. The clay minerals, such montmorillonite and illite, due to their high cation exchange capacity (CEC) and adsorption properties, can influence soil pH, electrical conductivity (EC), and organic matter stability, thereby affecting microbial accessibility to substrates and altering CH_4_ fluxes [22]. Furthermore, their ability to adsorb dissolved organic carbon and nutrients may also influence carbon turnover rates [23]. Given the complex interactions among soil amendments, microbial communities, and soil biogeochemical processes, understanding the effects of these amendments on CH_4_ emissions remains a key research challenge.

This study aimed to systematically evaluate the effects of different soil amendments, including microbial inoculants, biochar, humic acid, and montmorillonite, on methane emissions from paddy soil. A microcosm experiment was conducted to quantify cumulative CH_4_ and CO_2_ emissions and to examine changes in key soil physicochemical properties, including EC, pH, and acetate concentration. In addition, high-throughput 16S rRNA gene sequencing was used to assess shifts in microbial community structure, and functional genes related to methanogenesis (*mcrA*) and methane oxidation (*pmoA*) were quantified to explore the microbial mechanisms underlying CH_4_ emission responses. This study provides insights into how soil amendments influence greenhouse gas emissions and offers a basis for further research on sustainable rice cultivation practices.

## 2. Materials and Methods

### 2.1. Materials

Two commercial microbial inoculants, Duojun-360 (DJ) and Chabeijian (CB), were used to assess their effects on greenhouse gas emissions from paddy soil. DJ, purchased from Weifangjunyan Agricultural Technology Co., Ltd. (Weifang, China), is a complex microbial formulation containing *Bacillus subtilis*, *Bacillus licheniformis*, *Bacillus megaterium*, *Bacillus amyloliquefaciens*, and *Trichoderma harzianum*, with a viable cell count of at least 200 billion CFU g^−1^. CB, purchased from Shandong Lukanghaiboer Biotechnology Co., Ltd. (Jining, China), is a granular microbial inoculant composed of *Bacillus subtilis*, with a viable cell count of no less than 10 billion CFU g^−1^. Humic acid (HA) was obtained from Shanghai Macklin Biochemical Co., Ltd. (Shanghai, China). Biochar (BC) was synthesized by pyrolyzing crop straw at 500 °C for 2 h under a nitrogen atmosphere. Na-montmorillonite (NM), with a purity exceeding 99%, was purchased from Guzhang Shanlin Shiyu Mineral Products Co., Ltd. (Xiangxi, China).

### 2.2. Soil Sample Collection and Microcosm Incubation Experiment

Paddy soil was collected from Chashan Town, Dongguan City, Guangdong Province, China (23°02′ N, 113°45′ E). Soil samples were collected from the top 0–20 cm layer at five randomly selected sites and thoroughly homogenized to form a composite sample [22]. The composite soil was air-dried, passed through a 0.5 mm sieve, and stored at 4 °C before use. The anaerobic soil microcosm experiment was conducted in an anaerobic incubation system (Figure S1). The experiment included six treatments, each with four replicates: a control (CK, no amendment) and five amendment treatments, with each amendment applied at 10 g kg^−1^ soil. For each replicate, 10 g of prepared soil was placed into a sterile 100 mL serum bottle, and the corresponding amendment was thoroughly mixed with the soil. Then, 20 mL of water was added to achieve a water-to-soil ratio of 2:1 (*v*/*w*). The headspace was purged with nitrogen gas for 15 min to establish anaerobic conditions, with dissolved oxygen maintained at approximately 0.05 mg L^−1^ as measured using a DZS-708 L meter (INESA, Shanghai, China). Finally, the bottles were sealed and incubated in the dark at 30 ± 1 °C for 60 days.

### 2.3. Gas Emission and Soil Property Measurements

The concentrations of CH_4_ and CO_2_ in the headspace of serum bottles were monitored using GC-9800 gas chromatography (Shanghai Kechuang Sepu Instrument Co., Shanghai, China). Soil pH was measured by mixing soil with water at a 1:2.5 (*w*/*w*) ratio using a calibrated pH meter, while electrical conductivity (EC) was determined with a DDSJ-319 L conductivity meter (INESA, China). Soil acetate concentrations were determined by analyzing the filtered supernatant derived from the soil slurry using liquid chromatography (Shimadzu LC-SIL-16, Kyoto, Japan). [24].

### 2.4. Kinetic Analysis of Methanogenesis

The kinetics of Methane emission were evaluated using a modified Gompertz model as described by Equation [25].

$$P(t)=P_{max}*exp\left\{-exp\left[\frac{R_{m}*e}{P_{max}}*\left(\lambda -t\right)+1\right]\right\}$$

where P(t) is the cumulative methane yield at time t (μmol/g soil); $P_{max}$ is the maximum methane production potential (μmol/g soil); $R_{m}$ is the maximum methane production rate (μmol/g soil/d); λ is the lag-phase time (d); and e is 2.71828.

### 2.5. DNA Extraction and Illumina Amplicon Sequencing

After 60 days of anaerobic incubation, soil samples from the CK, DJ, and CB groups were selected for DNA extraction because the microbial inoculants showed stronger regulatory effects on CH_4_ emissions than the other amendments, with DJ and CB representing two contrasting methane emission responses. For each treatment, DNA was extracted from 4 independent biological replicates. The soil samples were freeze-dried, and genomic DNA was extracted using the E.Z.N.A. Soil DNA Kit (Omega Bio-Tek, Inc., Norcross, GA, USA). DNA quality and concentration were determined using a NanoDrop 2000 spectrophotometer (Thermo Fisher Scientific, Inc., Waltham, MA, USA).The V4 hypervariable region of the bacterial and archaeal 16S rRNA gene was amplified using the universal primers 515F (GTGCCAGCMGCCGCGGTAA) and 806R (GGACTACHVGGGTWTCTAAT) [26]. An 8 bp barcode sequence was added to the 5′ end of both the forward and reverse primers to distinguish among samples. PCR amplification was performed using an ABI 9700 PCR instrument (Applied Biosystems, Inc., Foster City, CA, USA) under the following conditions: initial denaturation at 95 °C for 5 min; 28 cycles of denaturation at 95 °C for 30 s, annealing at 55 °C for 50 s, and extension at 72 °C for 45 s; followed by a final extension at 72 °C for 10 min. The PCR products were purified using an Agencourt AMPure XP Kit (Beckman Coulter, Inc., Brea, CA, USA). Sequencing libraries were generated using the NEBNext Ultra II DNA Library Prep Kit (New England Biolabs, Inc., Ipswich, MA, USA) following the manufacturer’s instructions. Library quality was assessed using a NanoDrop 2000 spectrophotometer, an Agilent 2100 Bioanalyzer (Agilent Technologies, Inc., Santa Clara, CA, USA), and an ABI StepOnePlus Real-Time PCR System (Applied Biosystems, Inc., Foster City, CA, USA).

### 2.6. Quantitative PCR Analysis

Quantitative PCR (qPCR) was performed to quantify key functional genes involved in methane metabolism in soil samples from the CK, DJ, and CB groups after 60 days of anaerobic incubation. The qPCR analyses were carried out using an ABI 7500 thermocycler (Applied Biosystems, USA). The methyl-coenzyme M reductase (*mcrA*) gene was quantitatively analyzed using primers MLf (5′-GGTGGTGTMGGATTCACACARTAYGCWACAGC-3′) and MLr (5′-TTCATTGCRTAGTTWGGRTAGTT-3′) [27,28]. The particulate methane monooxygenase (*pmoA*) gene was quantitatively analyzed using primers (5′-GGNGACTGGGACTTCTGG-3′) and (5′-CCGGMGCAACGTCYTTACC-3′) [29,30]. The qPCR amplification conditions were as follows: initial denaturation at 94 °C for 5 min, followed by 30 cycles of denaturation at 94 °C for 30 s, annealing at 55 °C for 30 s, and extension at 72 °C for 30 s. A final extension was performed at 72 °C for 10 min [31]. Plasmid standards were serially diluted 10-fold increments from 10^1^ to 10^5^, and 2 μL of each dilution was used as a template to establish a standard curve. The correlation coefficient (R^2^) of the standard curve was ≥0.99, ensuring high accuracy and reliability of the qPCR assay.

### 2.7. Statistical Analysis

The experimental data were analyzed using Origin 2022 and R (version 4.3.1). Differences among treatment groups were evaluated using one-way analysis of variance (ANOVA), followed by Tukey’s post hoc test for pairwise comparisons when significant effects were detected. Principal component analysis (PCA) was performed on the KO–Bray–Curtis distance matrix using the vegan package, while alpha (α) and beta (β) diversity indices were calculated to evaluate microbial community diversity, with graphical representations generated via ggplot2 (version 3.4.3) [32].

## 3. Results

### 3.1. Effects of Soil Amendments on CH_4_ and CO_2_ Emissions from Paddy Fields

**Figure 1:**
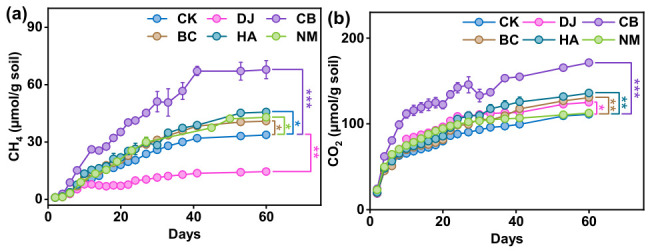
Cumulative CH_4_ (**a**) and CO_2_ (**b**) emissions from paddy soil over 60 days of anaerobic incubation under various soil amendments. Data are presented as means ± SD (*n* = 4). Significant differences between treatments are indicated by asterisks (* *p* < 0.05; ** *p* < 0.01; *** *p* < 0.001).

The cumulative methane emissions from paddy soil during 60 days of anaerobic incubation are presented in Figure 1a. In CK, cumulative CH_4_ emissions reached 33.8 ± 0.09 μmol/g soil, establishing the baseline for methane production. The DJ treatment significantly suppressed methane production, reducing cumulative CH_4_ emissions to 14.49 ± 0.71 μmol/g soil, which corresponds to a 57.13% decrease compared to CK. In contrast, the CB treatment markedly enhanced methane production, with cumulative CH_4_ emissions reaching 67.87 ± 4.76 μmol/g soil, corresponding to a 100.79% increase relative to CK. The BC, HA, and NM treatments produced more moderate increases, with cumulative CH_4_ emissions of 41.00 ± 1.26, 45.70 ± 0.82, and 42.89 ± 1.10 μmol/g soil, respectively, corresponding to increases of 21.30%, 35.21%, and 26.89% relative to CK. Moreover, the methane production curves for all treatments eventually reached a plateau, suggesting that the soil’s methanogenic potential approached saturation, possibly due to substrate limitations or shifts in microbial activity. The kinetics of methane production were analyzed using a modified Gompertz model (Table 1). In the CK treatment, the maximum methane production potential (P_max_) was 34.75 ± 0.89 μmol/g soil. The DJ treatment significantly reduced P_max_ to 14.85 ± 0.21 μmol/g soil, a substantial decrease compared to CK. In contrast, the P_max_ values for the CB, BC, HA, and NM treatments increased to 70.86 ± 3.07, 42.00 ± 1.00, 47.13 ± 2.04 and 43.06 ± 1.16 μmol/g soil, respectively, with the CB treatment exhibiting the greatest enhancement. Similarly, the maximum methane production rate (R_m_) followed this trend. In the DJ treatment, R_m_ decreased by 56.7% compared to CK (from 1.04 ± 0.05 to 0.45 ± 0.01 μmol/g soil/d), whereas R_m_ increased by 83.6, 24.03, 17.3 and 31.73% in the CB, BC, HA, and NM treatments, reaching 1.91 ± 0.13, 1.29 ± 0.05, 1.22 ± 0.08, and 1.37 ± 0.09 μmol/g soil/d, respectively. Moreover, the soil amendments also influenced soil CO_2_ emissions (Figure 1b). CK exhibited cumulative CO_2_ emissions of 111.28 ± 1.26 μmol/g soil after 60 days. In contrast, the CB, BC, HA and DJ treatments significantly increased CO_2_ emissions to 171.21 ± 0.94, 130.78 ± 4.92, 135.86 ± 1.71 and 125.19 ± 1.19 μmol/g soil, representing increases of 53.85, 17.52, 21.32 and 12.50%, respectively, compared to CK. Although the NM treatments showed higher CO_2_ emissions than CK during the early and mid-incubation stages, their cumulative emissions decelerated over time, reaching 112.70 ± 1.22 μmol/g soil after 60 days.

### 3.2. Effects of Soil Amendments on the Physicochemical Properties

**Figure 2:**
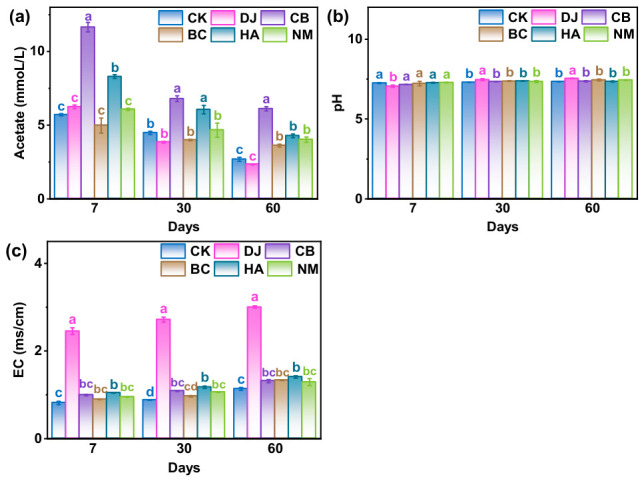
Changes in acetate concentrations (**a**), pH (**b**) and EC (**c**) in paddy soil under various soil amendments after 60 days of anaerobic incubation. Data are presented as means ± SD (*n* = 4). Different lowercase letters denote significant differences among treatments (*p* < 0.05).

The basic physicochemical properties of the soil used in this study were determined prior to the experiments. The soil exhibited a pH of 6.42, an electrical conductivity of 0.845 mS/cm, and a cation exchange capacity of 24.50 cmol/kg. Elemental analysis revealed concentrations of 36.5 g/kg for Fe, 20.7 g/kg for K, 3.25 g/kg for P, and 0.59 g/kg for S. In addition, the soil texture was characterized by a particle size distribution of 10.13% clay, 60.2% silt, and 29.67% sand. The application of soil amendments markedly altered acetate concentration, pH, and EC in paddy soil during anaerobic incubation (Figure 2). Acetate concentrations peaked during the early stage of incubation. On day 7, the DJ, CB, HA, and NM treatments showed higher acetate concentrations of 6.240 ± 0.129, 11.657 ± 0.319, 8.305 ± 0.126, and 6.078 ± 0.073 mmol/L, respectively, compared with CK (5.705 ± 0.083 mmol/L), whereas the BC treatment showed a lower acetate concentration of 4.976 ± 0.512 mmol/L. During incubation, acetate concentrations gradually declined by 53.1%, 62.4%, 47.6%, 27.5%, 48.6%, and 33.9% in the CK, DJ, CB, BC, HA, and NM treatments, respectively. Soil pH showed a modest increasing trend in all treatments. After 60 days, the pH values in the CK, CB, BC, HA, and NM treatments ranged from 7.34 to 7.44, whereas the DJ treatment increased to 7.55. Moreover, EC increased from 1.136 ± 0.003 mS/cm in CK to 1.290–1.409 mS/cm in the CB, BC, HA, and NM treatments, while the DJ treatment showed the highest EC value of 3.006 ± 0.026 mS/cm.

### 3.3. Effects of DJ and CB Amendments on Microbial Community

**Figure 3:**
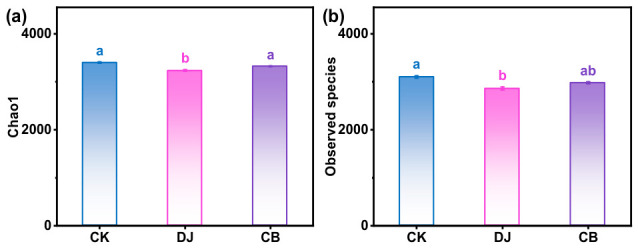

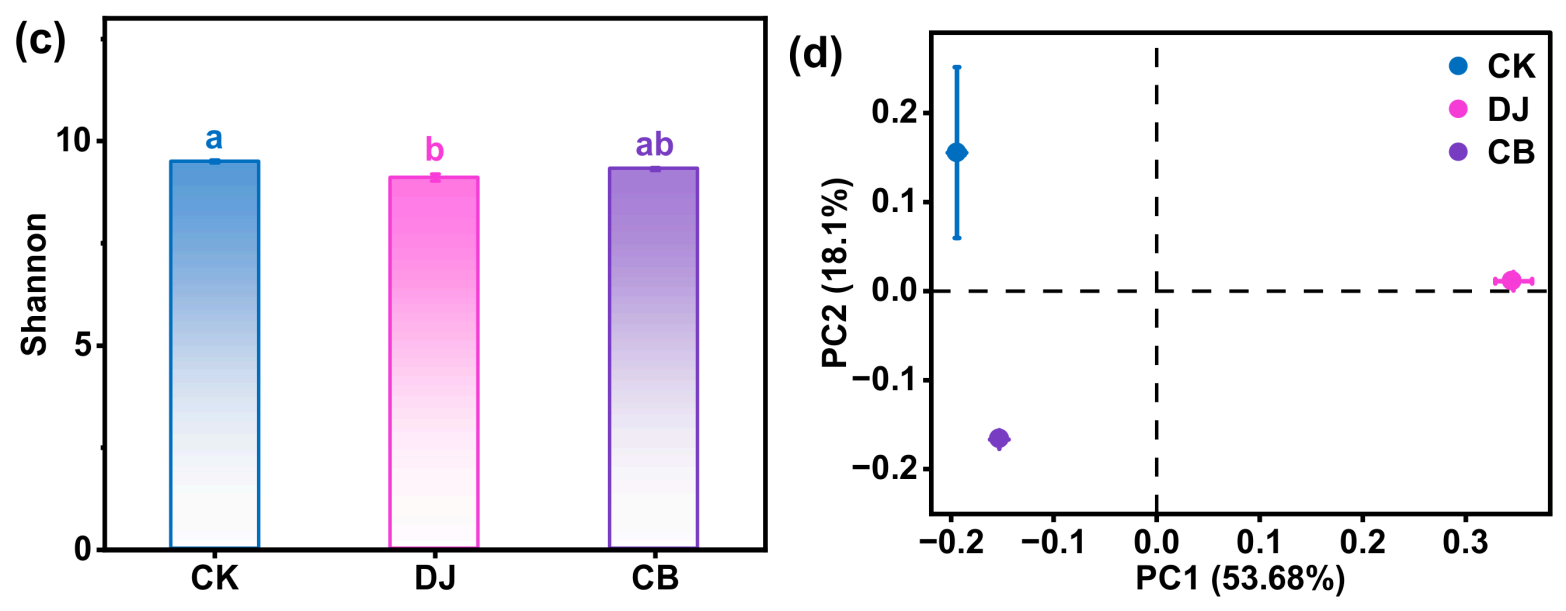
Bacterial community structure in paddy soil under DJ and CB amendments. Chao1 index (**a**), Observed species (**b**), Shannon index (**c**) and principal component analysis (PCA), constructed from the Bray–Curtis difference matrix, showing differences in bacterial communities between treatments (**d**). Data are presented as means ± SD (*n* = 4). Different lowercase letters denote significant differences among treatments (*p* < 0.05).

**Figure 4:**
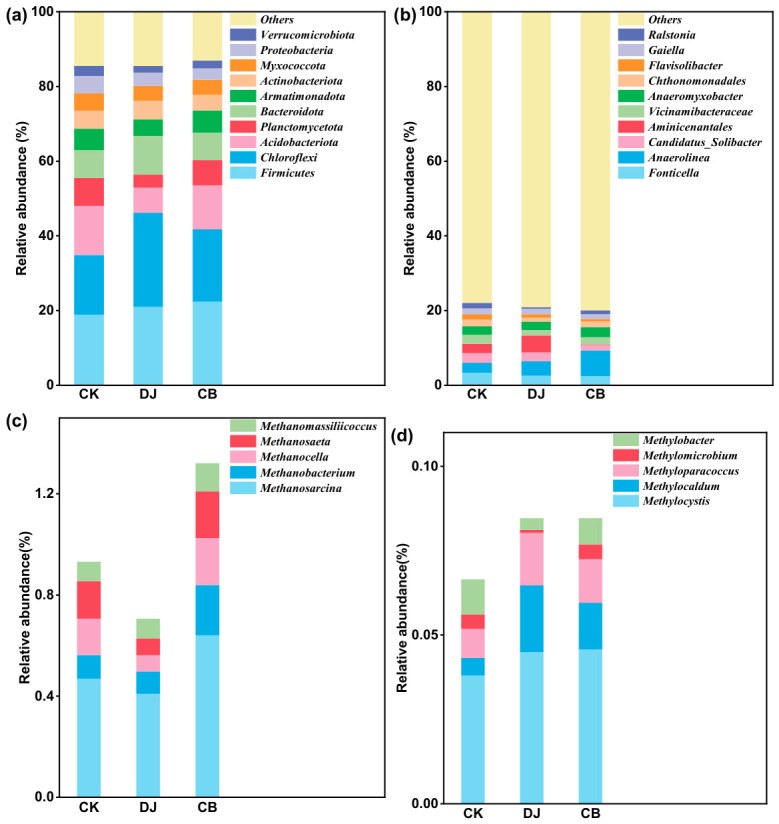
(**a**,**b**) Relative abundance of microbial communities at phylum (**a**) and genus (**b**) level in paddy soil under DJ and CB amendments compared to CK. (**c**,**d**) Relative abundance of methanogens (**c**) and methanotrophs (**d**) in paddy soil under DJ and CB amendments compared to CK.

High-throughput sequencing was performed to evaluate the effects of DJ and CB amendments on microbial richness, diversity, and community structure in paddy soil after 60 days of anaerobic incubation. Both DJ and CB treatments decreased the Chao1 index, observed species count, and Shannon index, with the DJ treatment showing a more pronounced decrease (Figure 3a–c). Principal component analysis (PCA) and permutational multivariate analysis of variance (*p* < 0.05) showed that the microbial community structures in the DJ and CB treatments were significantly separated from that of CK (Figure 3d). At the phylum level, *Firmicutes*, *Chloroflexi*, *Acidobacteriota*, and *Planctomycetota* were the dominant groups (Figure 4a). Compared with CK, the relative abundance of Firmicutes decreased in the DJ treatment but increased in the CB treatment. *Chloroflexi* increased in both DJ and CB treatments, with a more pronounced increase in the DJ treatment. *Acidobacteriota* decreased in the DJ treatment but increased in the CB treatment. At the genus level, the dominant genera included *Fonticella*, *Anaerolinea*, *Candidatus_Solibacter*, and *Aminicenantales* (Figure 4b). The CB treatment increased the relative abundance of *Fonticella* compared with CK, whereas the DJ treatment increased the relative abundance of *Anaerolinea*.

### 3.4. Effects of DJ and CB Amendments on Methanogenic and Methanotrophic Communities and Functional Genes

**Figure 5:**
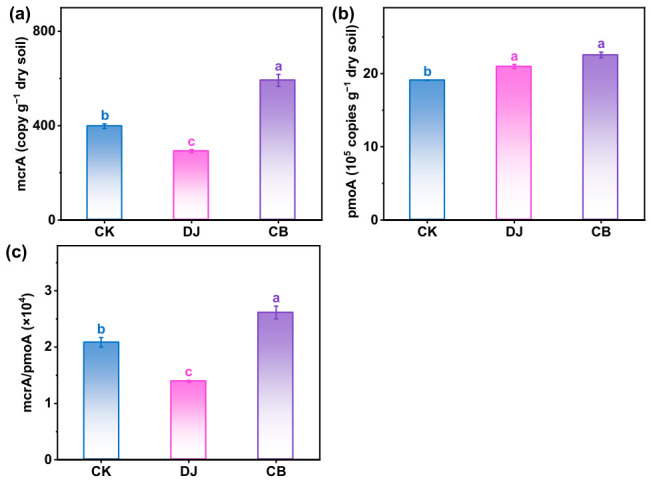
(**a**,**b**) The quantity of functional genes *mcrA* (**a**) and *pmoA* (**b**) in paddy soil under DJ and CB amendments. (**c**) The ratio of *mcrA*/*pmoA* for paddy soil under DJ and CB amendments (**c**). Data are presented as means ± SD (*n* = 4). Different lowercase letters denote significant differences among treatments (*p* < 0.05).

The effects of DJ and CB amendments on methane-cycling microbial communities and functional genes are shown in Figure 4c,d and Figure 5. At the genus level, the detected methanogens mainly included *Methanosarcina*, *Methanobacterium*, *Methanocella*, *Methanosaeta*, and *Methanomassiliicoccus* (Figure 4c). Compared with CK, the CB amendment increased the overall relative abundance of methanogens by 24.2%, whereas the DJ amendment decreased it by 42.0%. Among these genera, *Methanosarcina*, *Methanobacterium*, *Methanocella*, and *Methanosaeta* showed higher relative abundance under CB treatment but lower relative abundance under DJ treatment. In contrast, the relative abundance of methanotrophs increased by approximately 27% in both DJ and CB treatments compared with CK (Figure 4d). The dominant methanotrophic genera included *Methylocystis*, *Methylocaldum*, *Methyloparacoccus*, *Methylomicrobium*, and *Methylobacter*. The qPCR analysis further showed that the abundance of *mcrA* increased by 48.7% in the CB treatment but decreased by 26.9% in the DJ treatment (Figure 5a). The abundance of *pmoA* increased by approximately 18.1% and 9.8% in the CB and DJ treatments, respectively (Figure 5b). The *mcrA*/*pmoA* ratio increased in the CB treatment but decreased in the DJ treatment compared with CK (Figure 5c).

## 4. Discussion

### 4.1. Greenhouse Gas Emission and Soil Physicochemical Responses to Soil Amendments

The present study showed that soil amendments distinctly altered CH_4_ and CO_2_ emissions from anaerobic paddy soil microcosms. Among the five amendments tested, the two commercial microbial inoculants induced the most pronounced but opposite response in CH_4_ production. DJ significantly reduced cumulative CH_4_ emissions and methane production potential, whereas CB shown the significant increase in cumulative CH_4_ emissions, maximum methane production potential, and methane production rate. The BC, HA, and NM treatments also increased CH_4_ emissions, but their effects were more moderate than that of CB. In addition, most amendments increased cumulative CO_2_ emissions, with CB showing the enhancement. These results indicate that different soil amendments affected anaerobic carbon transformation in paddy soil to different extents, with DJ and CB representing two contrasting patterns of methane suppression and stimulation.

The amendments also induced distinct soil physicochemical responses, as reflected by changes in acetate concentration, pH, and EC. Acetate is a critical substrate for microbial anaerobic methane production [33]. The early peak in acetate concentration may have resulted from the rapid decomposition of abundant organic matter during the initial stage of anaerobic incubation. The decrease in acetate concentration in the CB treatment from day 7 to day 60 suggested enhanced acetate consumption under anaerobic conditions, which was consistent with methane and carbon dioxide production [33]. The increase in soil pH under DJ treatment may have contributed to the reduction in methane emissions, as most methanogens in paddy soil have an optimal pH close to neutrality [34]. In addition, the elevated EC observed in the DJ treatment may be attributed to its microbial composition, including *Bacillus subtilis*, *Bacillus licheniformis*, *Bacillus megaterium*, *Bacillus amyloliquefaciens*, and *Trichoderma harzianum*. Previous studies have reported that these microorganisms can secrete organic acids, such as citric and gluconic acids, which promote the dissolution and weathering of soil minerals and release additional cations, thereby increasing EC [35].

### 4.2. Microbial Community Restructuring Associated with Carbon Transformation

The decreases in the Chao1 index, observed species count, and Shannon index suggest that DJ and CB amendments altered the soil microbial community, thereby reducing overall species richness and diversity [36]. The PCA results further showed that the microbial community structures under DJ and CB treatments were clearly separated from that under CK, suggesting shifts in the overall community composition of paddy soil. The phylum-level changes also suggest that these amendments may be associated with differences in microbial processes related to carbon transformation. *Firmicutes* are commonly involved in the anaerobic degradation of complex organic matter, producing simpler compounds such as acetate, H_2_, and CO_2_, which may serve as substrates or intermediates for methanogenic processes [37]. Therefore, the increased relative abundance of Firmicutes under CB treatment was consistent with the enhanced CH_4_ and CO_2_ emissions observed in this treatment (Figure 1). The enrichment of Chloroflexi in both DJ and CB treatments may be related to complex organic matter degradation, and CO_2_ production, rather than directly indicating methane production [38]. *Acidobacteriota* are also involved in organic matter decomposition and carbon cycling by breaking down complex compounds into simpler molecules such as acetate, which may be related to the different acetate patterns observed between DJ and CB treatments [38]. At the genus level, the increased relative abundance of *Fonticella* under CB treatment may indicate enhanced anaerobic fermentation and acetate formation, potentially thereby providing more substrates for methanogens [39]. In contrast, DJ treatment increased the relative abundance of *Anaerolinea*, which is primarily involved in the degradation of complex organic compounds, although direct evidence linking this genus to methanogenesis remains limited [40,41]. Furthermore, the relative abundance of *Candidatus Solibacter*, a member of the *Acidobacteriota*, increased in CB-treated soils but decreased in DJ-treated soils, consistent with the phylum-level trend of *Acidobacteriota* [42]. In addition, *Aminicenantales* are known to participate in anaerobic degradation of complex organic matter and the production of short-chain fatty acids that can serve as substrates for methanogens [43]. Previous studies have also shown that *Aminicenantales* can establish metabolic networks with syntrophic bacteria and methanogens, potentially facilitating the conversion of organic matter into methane [44]. Overall, these results suggest that DJ and CB amendments reshaped the soil microbial community in different ways, which may be linked to their contrasting effects on methane emissions.

### 4.3. Microbial Methane-Cycling Responses

Microbial communities involved in methane cycling showed changes consistent with the contrasting CH_4_ emission. *Methanosarcina* plays an important role in acetoclastic and methylotrophic methanogenesis in paddy soils and can use substrates such as acetate and methylated compounds for methane production [45,46]. *Methanobacterium* and *Methanocella* are hydrogenotrophic methanogens that use H_2_ to reduce CO_2_ to CH_4_ [47,48], whereas *Methanosaeta* is an acetoclastic methanogen that metabolizes acetate to CH_4_ and CO_2_ [49,50]. Therefore, the enrichment of these methanogenic groups under CB treatment was consistent with the acetate dynamics and increased CH_4_ emissions, while their decrease under DJ treatment was consistent with reduced methane production. Methanotrophs are capable of oxidizing CH_4_ to CO_2_ and therefore play an important role in methane consumption [51].

The increased abundance of methanotrophs in both DJ and CB treatments suggests that methane oxidation potential also have increased, which might partly be related to the observed CO_2_ emissions. Among the methanotrophs, *Methylocystis* was dominant genus and is classified as a type II methanotrophs, which can survive under low-oxygen conditions and utilize CH_4_ and acetate as substrates [52,53]. The observed increase in abundance of *Methylocystis* under CB and DJ treatments suggests that these amendments may have provided conditions favorable for its growth and potential methane oxidation [54,55]. Type I methanotrophs generally respond to high methane concentrations and oxygenated microenvironments [56,57]. The enrichment of *Methylocaldum* and *Methyloparacoccus* under the CB amendment might be associated with increased methane availability, which could provide more substrates for these methanotrophs and potentially be linked to the elevated CO_2_ emissions through enhanced methane oxidation.

The qPCR results for functional genes involved in methane cycling further supported the observed shifts in methane production and oxidation potential. The *mcrA* gene encodes methyl-coenzyme M reductase, which is involved in the final step of methane production and is widely used as a functional marker of methanogenic potential [58,59,60,61,62]. The abundance of *mcrA* increased under CB treatment but decreased under DJ treatment, consistent with the changes in methanogen abundance and CH_4_ emissions. The *pmoA* gene encodes particulate methane monooxygenase, a key enzyme involved in the initial oxidation of methane to methanol [63,64]. The increase in *pmoA* abundance under both CB and DJ treatments suggests an increase in methane oxidation potential, which was consistent with the increased abundance of methanotrophs [65]. The *mcrA*/*pmoA* ratio further provided an indicator of the relative balance between methane production and methane oxidation [66]. The increased *mcrA*/*pmoA* ratio under CB treatment indicates a shift toward high methane production potential, whereas the decreased ratio under DJ treatment suggests a relatively lower methane production potential.

### 4.4. Environmental Implications and Limitations

These findings indicate that soil amendments can markedly regulate methane emissions from anaerobic paddy soils by altering soil physicochemical properties, microbial community structure, and microbial methane cycling. The contrasting effects of DJ and CB inoculants further suggest that microbial inoculants should be carefully evaluated before agricultural application, because different formulations may have opposite effects on methane mitigation. DJ showed potential for reducing CH_4_ emissions, whereas CB may increase the risk of greenhouse gas emissions under flooded soil conditions. However, this study was conducted using a controlled microcosm incubation system, which cannot fully represent the complex hydrological conditions, plant effects, and seasonal variations in field paddy soils. Therefore, future studies should validate these amendment effects under field conditions and evaluate their long-term impacts on methane emissions, soil fertility, and rice productivity.

## 5. Conclusions

This study demonstrated that soil amendments had distinct effects on greenhouse gas emissions from anaerobic paddy soil, with microbial inoculants showing greater effects than biochar, humic acid, and montmorillonite. Among the tested amendments, DJ markedly reduced cumulative CH_4_ emissions by 57.1%, whereas CB increased CH_4_ emissions by 100.8% and also caused the greatest increase in CO_2_ emissions. The contrasting effects of these two microbial inoculants were associated with changes in soil physicochemical properties and microbial methane cycling. CB was associated with changes in acetate dynamics, enriched methanogenic taxa, such as *Methanosarcina*, *Methanobacterium*, *Methanocella*, and *Methanosaeta*, and increased the abundance of the methanogenesis marker gene *mcrA*, suggesting enhanced acetoclastic and hydrogenotrophic methanogenesis. In contrast, DJ decreased methanogen abundance and *mcrA* abundance, indicating suppressed methanogenic potential. Although both DJ and CB increased relative abundance of methanotrophic taxa, including *Methylocystis*, *Methylocaldum*, and *Methyloparacoccus*, as well as *pmoA* abundance, which may be associated with the elevated CO_2_ emissions through methane oxidation, the increase in CH_4_ emissions under CB treatment suggests that the net methane response was mainly driven by enhanced methanogenic activity rather than methane oxidation potential. Overall, these findings indicate that microbial inoculants can regulate methane emissions from paddy soils in opposite directions depending on their effects on soil properties, microbial community structure, and the balance between methane production and oxidation. Future field experiments are needed to examine whether the amendment-induced shifts in methane-cycling microorganisms and CH_4_ emissions can be sustained throughout rice cultivation.

## Figures and Tables

**Table 1:** Summary of the kinetic parameters of methane production.

Amendments	Methane Production (μmol/g Soil)	P_max_ (μmol/g Soil)	R_m_ (μmol/g Soil/d)	λ (d)	R^2^
CK	33.8 ± 0.09	34.75 ± 0.89	1.04 ± 0.05	2.14 ± 0.71	0.989
DJ	14.49 ± 0.71	14.85 ± 0.21	0.45 ±0.01	1.48 ± 0.52	0.998
CB	67.87 ± 4.76	70.86 ± 3.07	1.91 ± 0.13	1.04 ± 1.17	0.978
BC	41.00 ± 1.26	42.00 ± 1.00	1.29 ± 0.05	2.87 ± 0.64	0.991
HA	45.70 ± 0.82	47.13 ± 2.04	1.22 ± 0.08	1.70 ± 1.09	0.981
NM	42.89 ± 1.10	43.06 ± 1.16	1.37 ± 0.09	4.16 ± 0.84	0.991

## Data Availability

Data will be made available upon request.

## Supplementary Materials

The following supporting information can be downloaded at: https://www.mdpi.com/article/10.3390/biology15110883/s1, Figure S1: Schematic diagram of the soil microcosm incubation experiment. Headspace gas samples were collected using a gas-tight syringe on days 2, 4, 6, 8, 9, 10, 12, 13, 14, 16, 18, 19, 20, 22, 23, 24, 27, 30, 33, 37, 41, 45, 50, 53, and 60, and subsequently analyzed by gas chromatography to determine CH_4_ and CO_2_ concentrations.
