# Supplementary material for: Responses of Methane Emissions to Different Soil Amendments in Paddy Soil: Soil Properties, Microbial Communities, and Functional Genes
Source: Biology (Basel). 2026 Jun 3;15(11):883. doi: 10.3390/biology15110883 (PMC13255583; doi:10.3390/biology15110883)
Supplement: Supplementary file 1 [file biology-15-00883-s001.zip › biology-4341407-supplementary.pdf]

Supplementary Information

Responses of Methane Emissions to Different Soil Amendments in Paddy Soil: Soil Properties, Microbial Communities, and Functional Genes

Qiong Wu†, Dalu Deng†, Yuwen Zhang, Weiwen Liang, Yifan Li, Yaping Zhang, Yi Wang*

Guangdong Key Laboratory of Environmental Catalysis and Health Risk Control, Guangzhou Key Laboratory Environmental Catalysis and Pollution Control, School of Environmental Science and Engineering, Institute of Environmental Health and Pollution Control, Guangdong University of Technology, Guangzhou 510006, PR China

* Corresponding author
Email: wangyiii@gdut.edu.cn
† These authors contributed equally to this work and share first authorship.

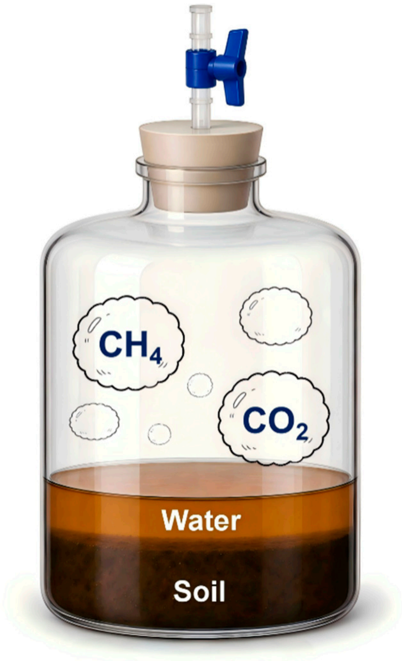

Figure S1 Schematic diagram of the soil microcosm incubation experiment. Headspace gas samples were collected using a gas-tight syringe on days 2, 4, 6, 8, 9, 10, 12, 13, 14, 16, 18, 19, 20, 22, 23, 24, 27, 30, 33, 37, 41, 45, 50, 53, and 60, and subsequently analyzed by gas chromatography to determine CH_4 and CO_2 concentrations.
